# MyomiRs as Markers of Insulin Resistance and Decreased Myogenesis in Skeletal Muscle of Diet-Induced Obese Mice

**DOI:** 10.3389/fendo.2016.00076

**Published:** 2016-06-27

**Authors:** Flávia de Toledo Frias, Mariana de Mendonça, Amanda Roque Martins, Ana Flávia Gindro, Bruno Cogliati, Rui Curi, Alice Cristina Rodrigues

**Affiliations:** ^1^Laboratory of Pharmacogenomics, Department of Pharmacology, University of Sao Paulo, Sao Paulo, Brazil; ^2^Laboratory of Cellular Physiology, Department of Physiology and Biophysics, Institute of Biomedical Sciences, University of Sao Paulo, Sao Paulo, Brazil; ^3^Department of Pathology, School of Veterinary Medicine and Animal Science, University of Sao Paulo, Sao Paulo, Brazil

**Keywords:** microRNA, insulin resistance, myogenesis, high-fat diet, skeletal muscle, IGF-1, myomiRs

## Abstract

High-fat diet (HFD) feeding causes insulin resistance (IR) in skeletal muscle of mice, which affects skeletal muscle metabolism and function. The involvement of muscle-specific microRNAs in the evolution of skeletal muscle IR during 4, 8, and 12 weeks in HFD-induced obese mice was investigated. After 4 weeks in HFD, mice were obese, hyperglycemic, and hyperinsulinemic; however, their muscles were responsive to insulin stimuli. Expressions of MyomiRs (miR-1, miR-133a, and miR-206) measured in soleus muscles were not different from those found in control mice. After 8 weeks of HFD feeding, glucose uptake was lower in skeletal muscle from obese mice compared to control mice, and we observed a significant decrease in miR-1a in soleus muscle when compared to HFD for 4 weeks. miR-1a expression continued to decay within time. After 12 weeks of HFD, miR-133a expression was upregulated when compared to the control group. Expression of miR-1a was negatively correlated with glycemia and positively correlated with the constant rate of plasma glucose disappearance. Pioglitazone treatment could not reverse decreases of miR-1a levels induced by HFD. Targets of myomiRs involved in insulin-growth factor (IGF)-1 pathway, such as *Igf-1, Irs-1, Rheb*, and follistatin, were reduced after 12 weeks in HFD and Mtor increased, when compared to the control or HFD for 4 or 8 weeks. These findings suggest for the first time that miR-1 may be a marker of the development of IR in skeletal muscle. Evidence was also presented that impairment in myomiRs expression contributes to decreased myogenesis and skeletal muscle growth reported in diabetes.

## Introduction

An imbalance between energy intake and energy expenditure can lead to obesity, which presents adipose tissue expansion and fat accumulation in non-adipose tissues, such as skeletal muscle, leading to the development of insulin resistance (IR) in this tissue (1). IR is typically found in type 2 diabetic subjects being characterized by impaired insulin action on adipocyte lipolysis, suppression of endogenous glucose production, and skeletal muscle glucose uptake. Since skeletal muscle constitutes about 40% of total body weight and accounts for about 80% of the insulin-stimulated postprandial glucose utilization (2), this tissue is the major player in the development of IR and type 2 diabetes (3). The treatment with insulin sensitizer can attenuate or even prevent the progressive loss of skeletal muscle mass and the establishment of sarcopenia in type 2 diabetic patients (4).

The myogenic regulatory factors (MRFs) that include myogenin, Murf4, Myf5, and MyoD, and members of the MEF2 family, essentially control skeletal muscle development (myogenesis) process. Various extracellular stimuli and distinct signaling pathways modulate expression of the MRFs. However, the insulin-growth factor (IGF)-1/phosphatidylinositol 3-kinase (PI3K)/AKT/MTOR is the main signaling pathway involved in skeletal differentiation and growth (5). IGF-1 binds to the IGF-1R, a tyrosine kinase receptor, and phosphorylates insulin receptor substrates (IRS) that activate PI3K. PI3K activation generates PIP3 that targets Akt, which mediates IGF-1 anabolic and anti-catabolic effects due *via* mTOR by inactivation of Foxo3 (6–8). Therefore, the IGF-1R signaling pathway plays a central role in muscle cell development.

MicroRNAs (miRNAs or miRs) are small, non-coding RNA molecules of ~18–24 that control gene expression (9). miRNAs regulate the stability and translation of conventional messenger RNAs by base pairing with the 3′ untranslated regions of protein-coding transcripts. Muscle-specific miRNAs, miR-1a, miR-133a, and miR-206, are recognized as important regulators of skeletal muscle development (10). miR-1 and miR-206 promoted differentiation of myoblasts through downregulation of HDAC4 and the p180 subunit of DNA polymerase alpha, while miR-133 promoted proliferation through downregulation of serum response factor (SRF) in C2C12 cells (11–13). Inhibition of endogenous miR-1 and miR-206 by using antimiRs blocked the downregulation of most targets in differentiating cells, thus indicating that miRNA activity and target interaction are required for muscle differentiation (14).

miR-133a represses IGF-1R expression and signaling pathway during skeletal myogenesis (15), suggesting it may be a potential therapeutic target in muscle diseases. On the other hand, IGF-1 and IGF-1R are targets for miR-1a, and a feedback loop between miR-1 expression and IGF-1 signal transduction cascade has been proposed in striated muscle (16). The role of IGF-1R on insulin signaling has been demonstrated in mice overexpressing a dominant-negative IGF-1 receptor (IGF-1R) in skeletal muscle (MKR mice) (17). These mice showed marked IR state and diabetes. Therefore, miRNA expression controlling IGF-1/PI3K/AKT pathway could well be marker of decreased myogenesis in soleus muscle of type 2 diabetic mice. The aim of this study was then to investigate the involvement of muscle-specific miRNAs in the evolution of skeletal muscle IR during 4, 8, and 12 weeks in high-fat diet (HFD)-induced obese (DIO) mice. Evidence was obtained that miR-1a may be an early-stage marker of IR evolution in skeletal muscle. Proliferation and differentiation are impaired in insulin resistant soleus muscles as miR-1a is consistently decreased. Increased expression of miR-133a (after 12 weeks of HFD feeding) was associated to transcriptional repression of IGF-1R and PI3K/AKT pathway in skeletal muscle of DIO mice.

## Materials and Methods

### Animals

One hundred and eleven male wild-type C57BL/6J mice (Jackson Laboratory, Bar Harbor, ME, USA) were maintained at 12:12-h light–dark cycle, 23 ± 2°C. They received a standard diet (Nuvilab-Nuvital Nutrients Ltd., Curitiba, Parana, Brazil) and water *ad libitum* until they were 8-week-old. Experimental Animal Ethics Committee of the Institute of Biomedical Sciences, University of Sao Paulo, approved the experimental protocol of this study (165/11/CEUA).

### Study Design

Sixty animals from the same brooding were randomly and equally divided into 2 groups (total of 15 per group): fed with balanced diet (CD) (9% kcal fat, 15% kcal protein, and 76% kcal carbohydrate) or with high-fat diet (HFD) (59% kcal fat, 15% kcal protein, and 26% kcal carbohydrate) (18). Animals received balanced (CD) or high-fat (HFD) diets for 4, 8, or 12 weeks. Next, we evaluated the effect of pioglitazone (35 mg/kg of b.w./day) on CD or HFD animals fed for 8 weeks. The animals were randomly divided into three groups (*n* = 7 per group): control diet (C), HFD (H), or HFD + plus pioglitazone (HP). Pioglitazone (EMS^®^) was mixed in the diet, and animals were treated for the last 2 weeks of the protocol. At the end of the defined period, the animals were fasted for 6 h and euthanized by cervical dislocation after ketamine intraperitoneal injection (100 mg/kg b.w.) and xylazine (50 mg/kg b.w.) for tissue harvesting. Soleus muscle was carefully dissected from the surrounding tissue, frozen in liquid nitrogen, and stored at −80°C until analysis being performed. Retroperitoneal, mesenteric, and epididymal adipose tissues were harvested and weighed to check adiposity level, and blood was collected for the determination of the metabolites. Body weight was determined every week and food intake every 3 days. The number of individual experiments was representative of at least three different litters. The total number of animals used in each experiment is indicated in the figure legends.

### Glucose Tolerance Test

After 4-, 8-, and 12-week feeding period, mice from all groups were fasted for 6 h and blood samples were collected from the tail vein for determination of fasting blood glucose (5 μL) and plasma insulin (20 μL) levels (time 0). After that, mice were given an intraperitoneal injection of glucose (2 g/kg b. w.) and blood samples were collected at 15, 30, 60, and 90 min afterwards. Blood glucose levels were measured using Glucose monoreagent Kit (Bioclin, Minas Gerais, Brazil). Insulinemia was measured by ELISA with EZRMI-13K kit (Millipore).

### Insulin Tolerance Test

The same initial procedure described above for Glucose tolerance test (GTT) was used. Mice received an intraperitoneal injection of insulin (0.75 mIU/g b.w.), and blood samples were collected at 0, 10, 20, and 30 min postinjection. Glycemia was measured using blood glucose test strips and a glucometer (Accu-Chek, Roche, USA). Insulin sensitivity was calculated using the plasma glucose disappearance rate (Kitt) during 10- to 30-min period (19).

### Glucose Uptake and Metabolism in Isolated Soleus Muscle

Soleus muscles from mice fed with HFD for 4–12 weeks were rapidly and carefully isolated and incubated as previously described, with minor modifications (20, 21). Intact soleus muscles were preincubated at 35°C in Krebs–Ringer bicarbonate buffer containing 5.6 mM glucose, pH 7.4, pre-gassed for 30 min with 95% O_2_/5% CO_2_ and agitation at 120 oscillations/min for 30 min. Subsequently, the muscles were transferred to the same buffer containing 0.3 μCi/mL d-[U-^14^C]glucose and 0.2 μCi/mL 2-deoxy-[2,6-^3^H]d-glucose and incubated under similar conditions for 1 h in the absence or presence of 10 mIU/mL insulin. Phenylethylamine, diluted 1:1 v/v in methanol, was added into a separate compartment for 14CO_2_ adsorption. After the incubation period, the muscles were briefly washed in cold buffer, dried on filter paper, and frozen in liquid N_2_. [14C]-Glycogen synthesis (as estimated by d-[14C]-glucose incorporation into glycogen), decarboxylation of d-[14C]-glucose, and 2-deoxy-d-[2,6-^3^H]-glucose uptake were measured as previously described (20).

### Total RNA Isolation

Total RNA was extracted from mice soleus muscle using miRVana RNA Isolation kit (Thermo Scientific, Waltham, MA, USA) according to the manufacturer’s instructions. Total RNA concentration was measured using the NanoDrop 2000 spectrophotometer (Thermo Scientific, Waltham, MA, USA). Purity was determined by the 260/280 nm ratio, and the value of 2.0 indicated a high purity of our samples.

### MicroRNA Expression

Relative miRNA expression was quantified by stem-loop RT-PCR technique described by Chen et al. (22). cDNA was synthetized from 10 ng total RNA extract according to the Taqman microRNA Assay Protocol by Life Technologies using Taqman microRNA Reverse Transcription kit (Thermo Scientific, Waltham, MA, USA) together with 50 nM TaqMan^®^ MicroRNA Assays (Thermo Scientific, Waltham, MA, USA). miRNA expression was then performed by the Taqman Real-time PCR method using the cDNA 15× diluted, 2× TaqMan Universal PCR master mix, and miRNA assays from Life Technologies: snoRNA-202 (001232), hsa-miR-1 (002222), hsa-miR-133a (002246), and hsa-miR-206 (000510). Real-time PCR was performed using ABI Prism 7500 equipment (Thermo Scientific, Waltham, MA, USA), following the universal protocol of amplification: 95°C for 10 min, 40 cycles of 95°C for 15 s, and 60°C for 1 min. The expression of miRNA relative to sno-202 (housekeeping) was determined using the 2^−ΔΔCT^ method.

### mRNA Expression

cDNA was synthetized from 500 ng total RNA extract using High Capacity cDNA Reverse Transcription Kit (Thermo Scientific) at 25°C for 10 min, 37°C for 120 min, with a final step of 5 min at 85°C in an Veriti Thermal Cycler (Thermo Scientific, Waltham, MA, USA). All PCR reactions was then performed using diluted (1/10) cDNA template, forward and reverse primers (200 nM each) and Power SYBR Green PCRMaster Mix (Thermo-Fisher). Real-time PCR was performed using Rotor Gene 3500 (Qiagen, Hilden Germany), following the universal protocol of amplification: 95°C for 10 min, 40 cycles of 95°C for 15 s, and 60°C for 1 min. To verify the purity of the products, a dissociation curve was performed after each run according to the manufacturer’s instructions. For the relative gene expression quantification, also determined by 2^−ΔΔCT^, 36b4 was used as reference gene, as 36b4 expression did not vary between diets or along the time. Genes analyzed included IGF-1 (*Igf-1*) and Igf-1 receptor (*Igf-1R*), insulin receptor substrate-1 (*Irs-1*), Ras homolog enriched in brain (*Rheb*), mechanistic target of rapamycin (serine/threonine kinase) (*mTOR*), regulatory-associated protein of mTOR complex 1 (*Rptor*), independent companion of mTOR complex 2 (*Rictor*), myogenic differentiation 1 (*Myod1*), myogenic factor 5 (*Myf5*), Myostatin (*Mstn*), Follistatin (*Fst*), Follistatin-like 1 (*Fstl1*), and histone deacetylase 4 (*HDAC4*). Primers’ sequences are shown in Table 1.

**Table 1:** **Primers’ sequences used to quantify mRNA expression**.

Gene	Seq forward	Seq reverse
*Igf-1*	GTGAGCCAAAGACACACCCA	ACCTCTGATTTTCCGAGTTGC
*Igf-1R*	CTCTGTTACCTCTCCACCAT	CTTCTCACACATGGGCTTCT
*Irs-1*	CTCAGTCCCAACCATAACCAG	GAGTGTTCATAGGCGAGATGG
*Rheb*	CGA TCC AAC CAT AGA GAA CAC	AAT ATT CAT CCT GCC CCG CT
*Mtor*	TGC CGC TGA GAG ATG ACA ATG	GTT GTT AAT GCT GAT GAG GG
*Rictor*	TCG GGG TTC GTG GTT CAT TA	GTC CTG TTT TGT TCC ACT GC
*Rptor*	ACC TGT TCA CAT CCT GCC TCA	GGC CAG GGA TCT TTT CTA TC
*Myod1*	GCCCGCGCTCCAACTGCTCTGAT	CCTACGGTGGTGCGCCCTCTGC
*Myf5*	TGACGGCATGCCTGAATGT	GCTGGACAAGCAATCCAAGC
*Fst*	AAAACCTACCGCAACGAATG	GGTCTGATCCACCACACAAG
*Fstl1*	AATGGCAAGACCTACCTCAACC	GTGCCCATCATAATCAACCTGG
*Mstn*	GCAAAATTGGCTCAAACAGCC	AGGGATTCAGCCCATCTTCTC
*Hdac4*	TGAGAGTGAGGAGGAAGAAGCG	CAAATGACACAGGGATGCCAG

*Igf-1, insulin-like growth factor 1; Igf-1R, insulin-like growth factor 1 receptor; Irs-1, insulin receptor substrate-1; Rheb, Ras homolog enriched in brain; Mtor, mechanistic target of rapamycin (serine/threonine kinase); Rptor, regulatory-associated protein of mTOR complex 1; Rictor, independent companion of MTOR complex 2; Myod1, myogenic differentiation1; Myf5, myogenic factor 5; Fst, follistatin; Fstl1, follistatin-like 1; Mstn, myostatin; Hdac4, histone deacetylase 4*.

### Bioinformatic Analysis

MyomiRs target prediction was performed using DIANA-microT-CDS algorithm v5.0 (23, 24). A score threshold of 0.7 was set for searches of miRNA recognition elements (MRE) located in both 3′UTR and CDS regions of mRNAs. Then, we used the DIANA-miRPath v2.0, an online software suite dedicated to the assessment of miRNA regulatory roles and the identification of controlled pathways. (25).

### Statistical Analysis

The results are presented as mean ± SEM and were analyzed by using one-, two-, or three-way ANOVA, followed by Bonferroni posttest. For two- and three-way ANOVA, when there was no interaction between the two or three factors, respectively, the factors or the interaction between two factors were considered separately, and the all pairwise multiple comparison test Holm–Sidak was performed. Spearman’s or Pearson’s rank correlation coefficient was used to find which variables were correlated. Then, a multivariate linear regression analysis was used to evaluate which variables could independently predict increments in glycemia. Variables that presented multicollinearity among the independent variables were excluded. Statistical tests were performed using the Sigma Stat version 3.5. Level of significance was set to *p* < 0.05.

## Results

### Body Weight Gain, Weight of Adipose Depots, and Glucose and Insulin Tolerance Tests

**Figure 1:**
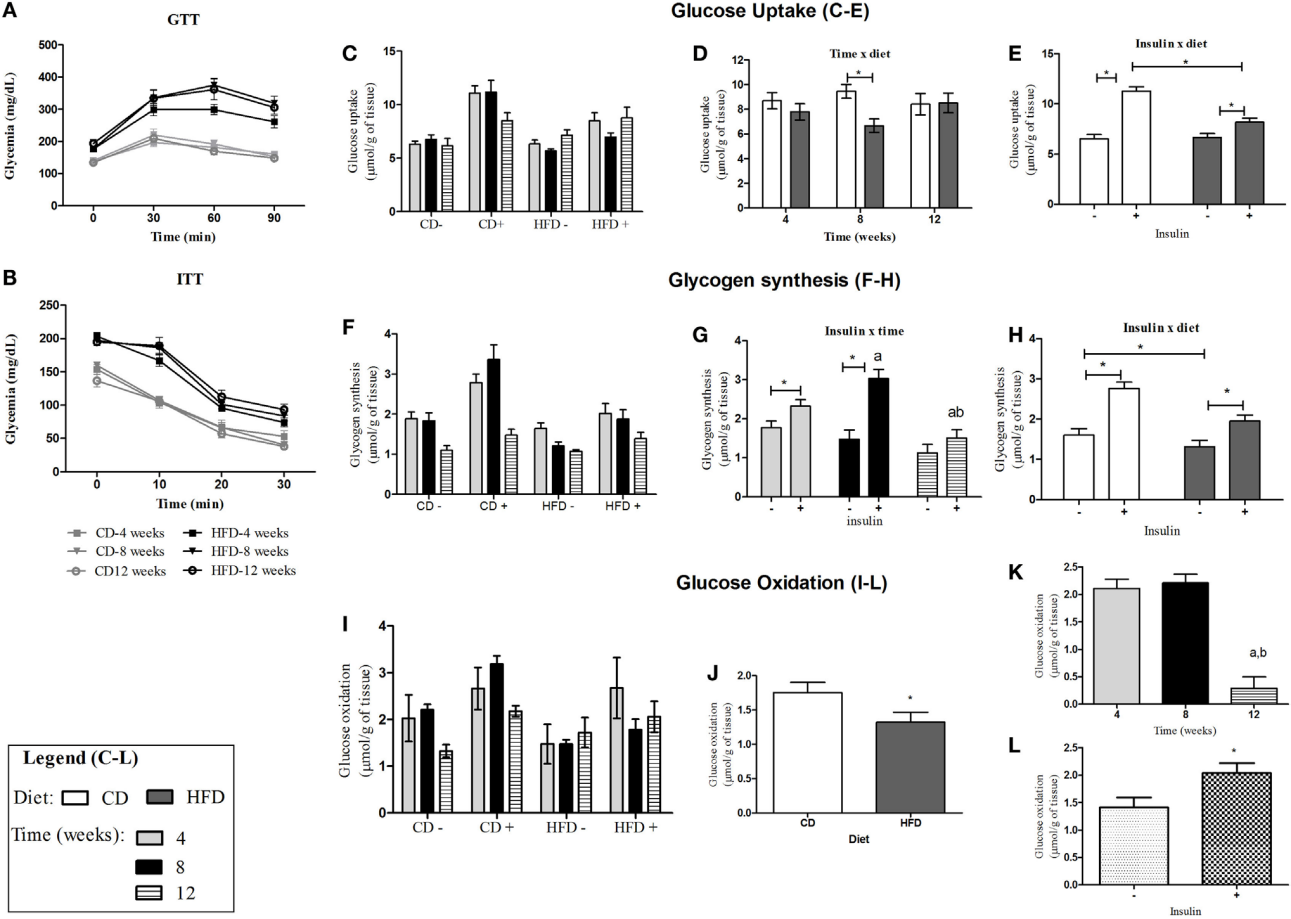
**Insulin sensitivity and glucose tolerance in control- or HFD-fed C57BL/6J mice**. **(A)** Glycemic curves obtained from GTT after injection of glucose (2 g/kg/b.w.); **(B)** glycemic curves obtained from ITT after injection of insulin (0.75 mIU/g b.w.); **(C,F,I)** glucose metabolism in soleus muscle of control- and HFD-fed animals after stimulation (+) or not (−) with insulin 10 mIU/mL after 4-, 8-, and 12-week feeding period. **(C–E)**: glucose uptake average for each group in the absence/presence of insulin **(C)**, interaction between insulin and diet **(D)**, and interaction between time and diet **(E)**. **(F–H)**: glycogen synthesis average for each group in the absence or presence of insulin **(F)**, interaction between insulin and time **(G)**, and interaction between insulin and diet **(H)**. **(I–L)**: glucose oxidation average for each group in the presence and absence of insulin **(I)** and effect of diet **(J)**, time **(K)**, and insulin **(L)** separately on glucose oxidation. a,b and *p < 0.05 as indicated by three-way ANOVA followed by all pairwise multiple comparison test Holm–Sidak for interaction between two factors or effect of just one factor. (a,b) compared to 4 or 8 weeks, respectively. CD, control-fed mice; HFD, high-fat diet-fed mice.

Mice fed with HFD showed augment of body weight gain, weights of adipose depots, fasting insulin levels, HOMA-IR, constant of glucose disapearance (Kitt), the area under the curve (AUC) of GTT, and so IR and glucose intolerance conditions were established already after 4 weeks (Table 2; Figures 1A,B). As expected, a prolonged time on HFD worsened the obesity and IR. Interestingly, after 8 and 12 weeks of HFD, gastrocnemius muscle weight was lower than that of CD mice (Table 2). We also noted a progressive increase in liver weight with time, which was a result of ectopic triglyceride (TG) accumulation as indicated by measurement of steatosis score, calculated after histopathological examination, and TG content (Table 2).

**Table 2:** **Adipose tissue weight, glucose, and insulin tolerance tests in mice fed either control or high-fat diets for 4, 8, or 12 weeks**.

Measurements	4 weeks		8 weeks		12 weeks		Time and diet	Time	Diet
	
	CD (15)	HFD (14)	CD (12)	HFD (15)	CD (13)	HFD (15)	p*	*p*	*p*
Initial body weight (g)	21.5 ± 1.5	22.3 ± 1.5	22.3 ± 0.13	22.0 ± 1.2	22.8 ± 1.7	22.3 ± 2.0	>0.05	>0.05	>0.05
Final body weight (g)	22.8 ± 1.2	**27.2 ± 2.3^a^**	23.6 ± 1.2	**32.1 ± 3.2^ab^**	24.1 ± 1.6	**36.1 ± 4.7^abc^**	**<0.05**	**–**	**–**
Body weight gain _(final−initital)_ (g)	1.3 ± 0.9	**4.9 ± 2.0^a^**	1.3 ± 1.2	**10.2 ± 3.11^ab^**	0.73 ± 1.83	**14.0 ± 3.3^abc^**	**<0.05**	**–**	**–**
Retroperitoneal fat pad (g)	0.11 ± 0.02	**0.30 ± 0.10^a^**	0.13 ± 0.04	**0.55 ± 0.16^ab^**	0.13 ± 0.03	**0.60 ± 0.14^ab^**	**<0.05**	**–**	**–**
Epidydimal fat pad (g)	0.37 ± 0.04	**0.71 ± 0.25^a^**	0.39 ± 0.08	**1.31 ± 0.33^ab^**	0.38 ± 0.09	**1.67 ± 0.41^abc^**	**<0.05**	**–**	**–**
Mesenteric fat pad (g)	0.30 ± 0.08	**0.44 ± 0.12**	0.25 ± 0.11	**0.45 ± 0.13**	0.21 ± 0.10	**0.48 ± 0.15**	>0.05	>0.05	**<0.05**
Gastrocnemius weight (mg)	127 ± 10	**144 ± 13**	139 ± 12	**150 ± 13**	**141 ± 13**	**156 ± 12**	>0.05	**<0.05**	**<0.05**
Relative gastrocnemius weight (mg/b.w.)	5.6 ± 0.4	5.4 ± 0.4	5.9 ± 0.5	**4.7 ± 0.3^ab^**	5.9 ± 0.6	**4.3 ± 0.5^ab^**	**<0.05**	**–**	**–**
Liver weight (g)	0.92 ± 0.14	0.87 ± 0.09	0.92 ± 0.09	**1.01 ± 0.12^b^**	0.91 ± 0.08	**1.08 ± 0.18^ab^**	**<0.05**	**–**	**–**
Liver TG content (g)	1.01 ± 0.58	0.73 ± 0.16	1.05 ± 0.84	1.82 ± 1.15	**4.97 ± 2.38**	**7.36 ± 3.57**	>0.05	**<0.05**	>0.05
Steatosis score (0–3)	0 (0–1)	0 (0–1)	0 (0–1)	**1 (0–1)^a^**	0 (0–1)	**1 (0–2)^ab^**	**<0.05**	**–**	**–**
Fasting glycemia (mg/dL)	136 ± 24	**177 ± 31**	144 ± 9	**188 ± 31**	126 ± 20	**168 ± 26**	>0.05	>0.05	**<0.05**
Fasting insulin levels (ng/mL)	0.41 ± 0.09	**0.99 ± 0.48**	0.30 ± 0.09	**1.24 ± 0.64**	**0.69 ± 0.54**	**1.83 ± 1.03**	>0.05	**<0.05**	**<0.05**
Kitt (%/min)	5.3 ± 0.51	**4.26 ± 0.71**	**4.99 ± 0.66**	**3.38 ± 0.98**	**4.66 ± 0.88**	**2.84 ± 0.74**	>0.05	**<0.05**	**<0.05**
HOMA-IR	2.9 ± 1.6	**7.7 ± 3.5**	1.7 ± 0.9	**10.8 ± 6.4**	2.4 ± 1.7	**16.0 ± 13.1**	>0.05	>0.05	**<0.05**
HOMA-β	50.3 ± 18.7	58.5 ± 28.7	28.3 ± 11.8	**87.6 ± 41.4^ab^**	44.6 ± 24.0	**122.7 ± 35.1^abc^**	**<0.05**	**-**	**-**
AUC GTT	15532 ± 3078	**24518 ± 4765**	16770 ± 2651	**28764** ± **4920**	15616 ± 2429	**26538 ± 6829**	>0.05	>0.05	**<0.05**

*AUC GTT, area under curve of glucose tolerance test; Kitt, constant for glycemia decay obtained from insulin tolerance test; HOMA, homeostasis model assessment; IR, insulin resistance; β, beta pancreatic cells. Data are expressed as mean ± SD, except for steatosis score, which is presented as medians with range (0: <5%; 1: 5–33%; 2: 33–66%; 3: >66% of hepatocytes with lipids). In bold, significant values of time or diet. In parenthesis, the total number of mice studied*.

*p for interaction of time and diet as indicated by two-way ANOVA.

*^a,b,c^p < 0.05 as indicated by Bonferroni posttest vs. control diet^a^; 4 weeks of HFD^b^; and 8 weeks of HFD^c^*.

Glucose metabolism and insulin responsiveness were investigated in soleus muscle that is rich in oxidative (I and IIa type) fibers (26) (Figures 1C–L). Insulin stimulated 2-DG uptake (Figure 1E), glycogen synthesis (Figure 1H), and glucose oxidation (Figure 1L) in soleus muscle of mice. Mice fed with HFD presented lowered 2-DG uptake (Figure 1E) and glycogen synthesis (Figure 1H) induced by insulin and glycogen oxidation (Figure 1J). After 8 weeks of HFD, soleus muscle presented significant decrease in glucose uptake (Figure 1D) compared to control-fed mice. After 12 weeks of feeding CD or HFD (20 weeks of age), soleus muscles were not responsive to insulin stimulus for glycogen synthesis anymore, and 20-week-old animals were less responsive to insulin-stimulated glycogen synthesis than 12-week-old or 16-week-old animals (fed for 4 or 8 weeks with CD or HFD, respectively) (Figure 1G). As well as this, soleus muscles from 20-week-old mice presented lower glucose oxidation, independently of the diet received (12 weeks of CD or HFD) (Figure 1K).

### miRNAs Expression in the Soleus Muscle of Mice Fed HFD for 4, 8, or 12 Weeks

**Figure 2:**
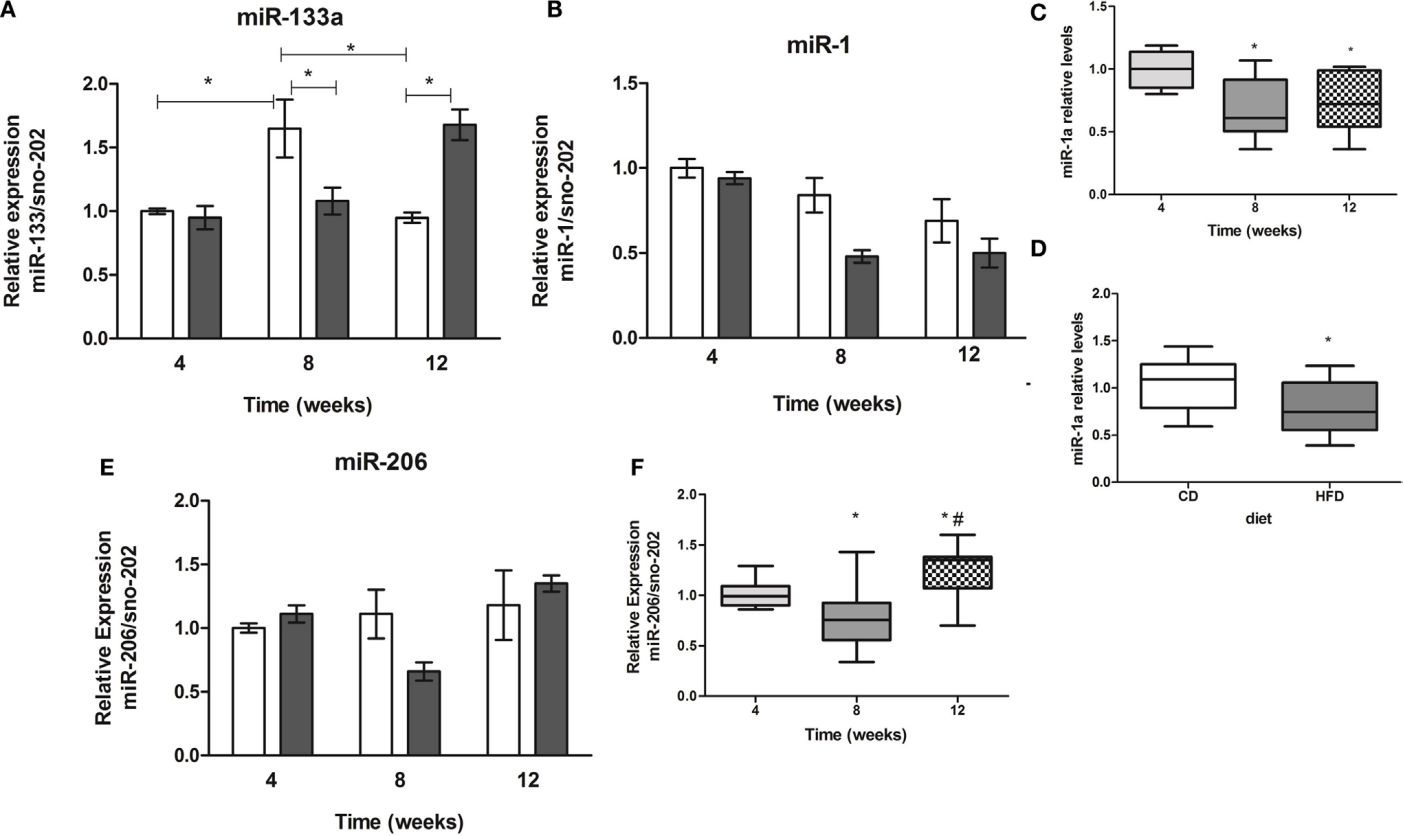
**Time-course of muscle-specific microRNAs miR-133a (A), miR-1a (B), and miR-206 (E) expression in soleus muscle of high-fat diet (HFD) and control diet (CD)-fed mice**. Total RNA was extracted from soleus muscle of HFD- and CD-fed mice, and 10 ng was used for stem-loop reverse transcription. RT products were used for TaqMan real-time PCR. SnoRNA-202 was used for normalization. *p < 0.05 as indicated by two-way ANOVA followed by Bonferroni posttest; **(C,D)** effect of diet and time separately on miR-1a expression, respectively, *p < 0.05 as indicated by one-way ANOVA followed by Holm–Sidak method; **(F)** effect of the time on miR-206 expression, *p < 0.05 as indicated by one-way ANOVA followed by Holm–Sidak method.

Two-way ANOVA revealed an interaction of period of time studied and diet only in miR-133a expression (Figure 2A). Different from control mice, animals on HFD showed an increase by twofold of miR-133a expression in soleus muscles after 12 weeks (Figure 2A). miR-1a expression was different along the time (4 vs. 8 or 12 weeks) and between control and HFD (Figures 2B–D), and miR-206 expression was different along the time (4 vs. 8 or 12 weeks, and 8 vs. 12 weeks, *p* < 0.05) (Figures 2E,F).

### Expression of mRNAs in Soleus Muscle of Mice fed HFD for 4, 8, and 12 Weeks

**Figure 3:**
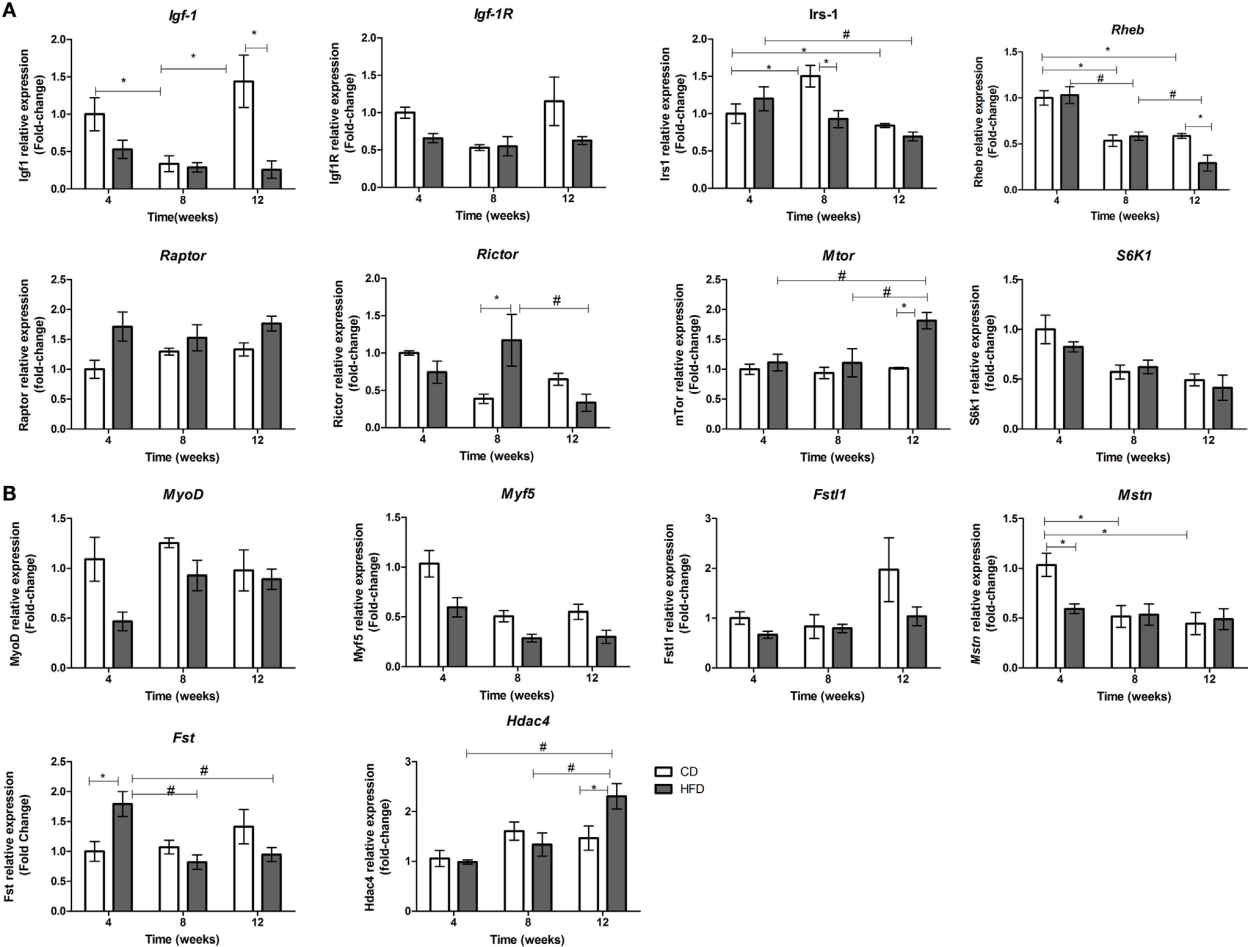
**Time-course of mRNAs expression involved in IGF-1/PI3K/AKT/MTOR pathway (A) and with myogenesis (B) in soleus muscle of high-fat diet (HFD)- and control diet (CD)-fed mice**. Total RNA was extracted from soleus muscle of HFD- and CD-fed mice, and 500 ng was used for reverse transcription. RT products were used for SyBR green real-time PCR. 36B4 gene was used for normalization. *p < 0.05 as indicated by two-way ANOVA followed by all pairwise multiple comparison procedure: Holm–Sidak method. Only significant values for interactions of time and diet are shown. For separate analysis of time and diet, please see the Section “Results.”

Due to the fact that myomiRs are strongly associated to myogenesis and myomiR targets are involved in IGF-1/PI3K/AKT/MTOR pathway, we postulated that decreases in miR-1a and increases in miR-133a levels could be associated with decreased myogenesis in skeletal muscles of insulin-resistant mice. Interaction between period of time studied and diet was found in mRNA expression of *Igf-1, Irs-1, Rheb, mTOR*, and *Rictor* (Mtorc2). HFD-fed mice showed a significant reduction on expression of *Igf-1, Igf-1R*, and *Rheb* after 12 weeks when compared to the paired control group (Figure 3A). This pattern of expression is consistent with an increase of miR-133a levels (Figure 2A), which has *Igf-1R* as target (15). *Irs-1* level was significantly lowered in soleus muscles in 8-week HFD-fed animals as compared with paired control and 4-week HFD mice. *mTOR* level was upregulated in soleus muscles of 12-week-fed HFD mice, which is consistent with Rheb inhibition. Mtorc1 (Rptor) was higher expressed in soleus muscle of HFD animals (CD: 1.2 ± 0.09 vs. HFD: 1.5 ± 0.12, *p* < 0.05), but it was not different from control after 12 weeks of HFD. This is in agreement with Mtorc1 upregulation in obesity (27). Levels of *Rictor* (mTorc2) increased only at 8 weeks of HFD when compared to control, and then is reduced after 12 weeks of HFD (Figure 3A). Mtorc2 phosphorylates AKT at residue Ser-473, activating it, and reduction in Rictor expression inhibits AKT (28). These results suggest IGF-1 anabolic effect is reduced in 12-week HFD mice. In order to confirm a decreased myogenesis in soleus muscle, we have measured some myogenic response factors. *Fst, Mstn*, and *Hdac4* expression showed an interaction between time and diet. Myostatin (*Mstn*) was lowered in 4-week HFD-fed animals as compared to the control group. Concurrently, its inhibitor Follistatin (*Fst*) was upregulated in soleus muscles of mice fed HFD for 4 weeks (Figure 3B). *Hdac4*, a transcription repressor, and target of miR-1 and miR-206, was increased in soleus muscle of mice in HFD for 12 weeks compared to control and with 4 and 8 weeks of HFD (Figure 3B). Expressions of *Myf5* was significantly reduced by HFD feeding (CD: 0.69 ± 0.08 vs. HFD: 0.39 ± 0.05, *p* < 0.05) and by the time (4 weeks: 0.81 ± 0.11 vs. 12 weeks: 0.41 ± 0.06, *p* < 0.05), independently. These observations are in agreement with the loss of skeletal muscle mass observed in sarcopenia, which is accelerated by diabetes. Interestingly, relative to body weight, a progressive loss of skeletal muscle was observed in HFD mice, especially at 8- and 12-weeks times, which was not observed in control (Table 2).

### Lower Expression of miR-1 is Correlated with Insulin Resistance in DIO Mice but Is Not Restored by Pioglitazone Treatment

**Figure 4:**
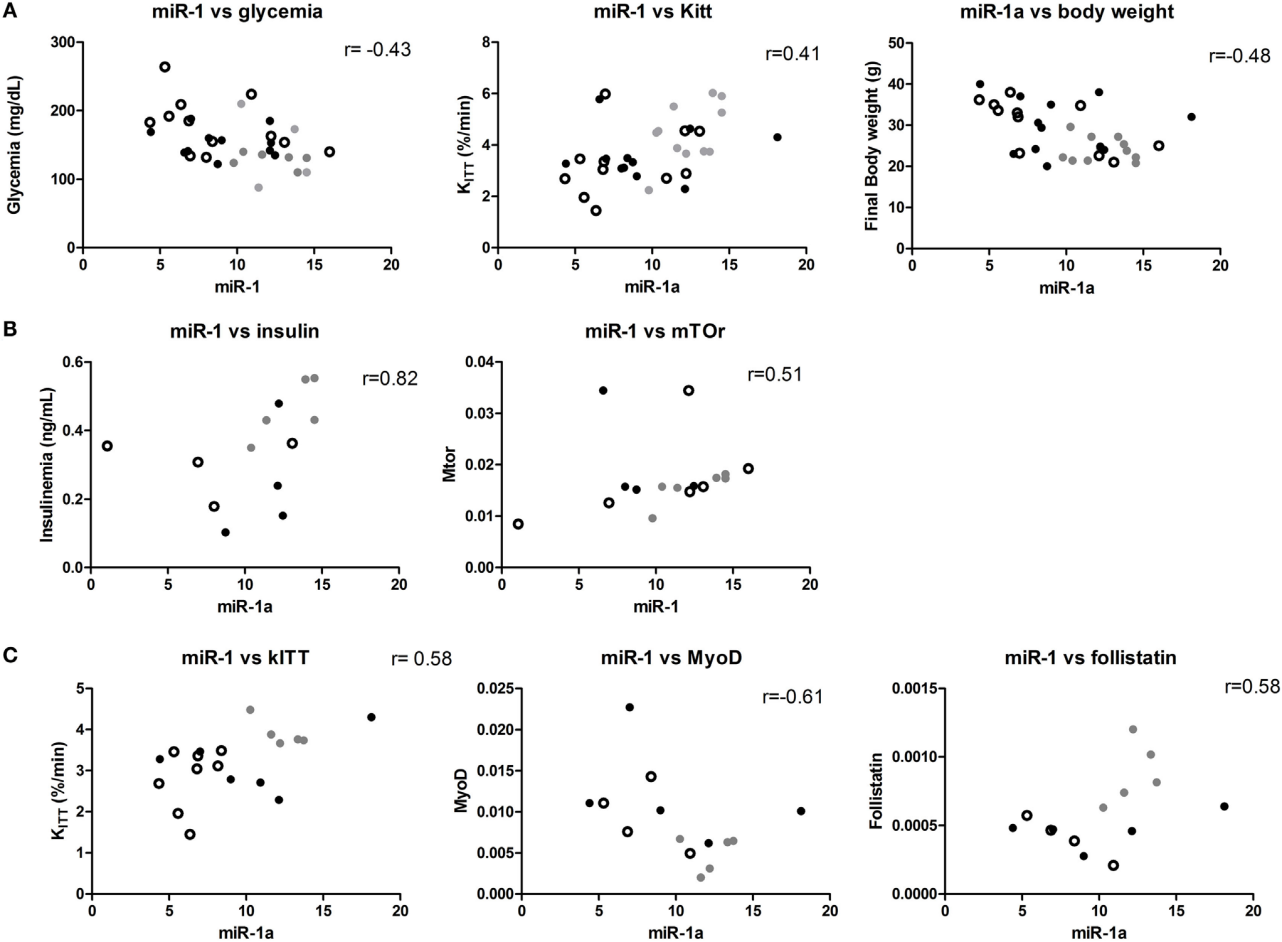
**miR-1 is correlated with metabolic parameters in mice**. **(A)** Samples from control and obese mice: significant correlations between miR-1, Kitt, and glycemia are shown; **(B)** only control mice samples were used for this analysis: insulin plasma levels and *mTor* mRNA significantly correlated with miR-1a levels; and **(C)** only obese mice were used in these analyses: significant correlations were found between miR-1a and Kitt, *MyoD* and *Fst* mRNA. Spearman’s correlation analysis was used to determine the correlation. Gray dot: 4 weeks, open circle: 8 weeks, and black dot: 12 weeks.

A significant positive correlation between miR-1 and miR-206 expression was found in all individuals (*r* = 0.70, *p* < 0.05). A negative correlation between miR-1 and fasting glycemia (*r* = −0.43, *p* < 0.05) and body weight (*r* = −0.48, *p* < 0.05) and a positive correlation between miR-1 and Kitt (*r* = 0.41, *p* < 0.05) were also found (Figure 4). This correlation was not observed on miR-206 levels (data not shown). When we separated control from obese, we found a strong positive correlation between plasma insulin concentration and miR-1 levels (*r* = 0.82, *p* < 0.05) and miR-1 expression and mTOR mRNA levels (*r* = 0.51, *p* < 0.05) in soleus muscles of control diet-fed mice. Interestingly, in C2C12 differentiating cells, Mtor is important for upregulation of MyoD, which in turn upregulates miR-1 (29). Mtor and miR-1 correlation was lost, but Kitt and *Fst* mRNA levels were positively correlated and body weight and *Myod* mRNA levels negatively correlated with miR-1 levels in soleus muscles (Figure 4).

Finally, we performed a multiple linear regression analysis, to understand which variables were independently associated to glycemia in our DIO model. As this model is very sensitive to body weight, this variable was used as an independent variable. Body weight was correlated with glycemia, liver weight, Steatosis score, *Rheb* and *Igf-1* expression, and gastrocnemius weight (data not shown). Other variables used in the model were miR-1a (correlated to glycemia) and miR-206 (correlated to miR-1a levels), and miR-133a, *Rheb, Igf-1, Mtor*, and *Mstn* mRNAs expression, and gastrocnemius weight, because they were significantly associated to IR progression. The model with an adjusted *R^2^* = 0.734 resulted in the following variables appearing to account for the ability to predict glycemia (*p* < 0.05): body weight, gastrocnemius weight, *Mtor, Rheb, Mstn*, miR-1a, and miR-206 expression.

**Figure 5:**
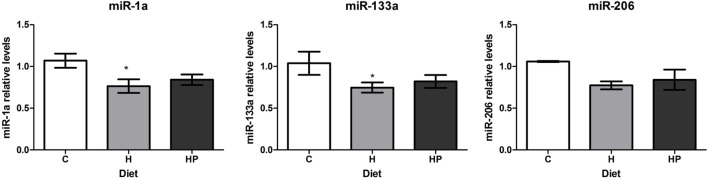
**Effect of pioglitazone 35 mg/kg/day on muscle-specific microRNAs expression in soleus muscles of HFD-fed mice**. Animals were fed for 8 weeks with high-fat diet and treated with pioglitazone for 2 weeks. Total RNA was extracted from soleus muscles for microRNA expression. *p < 0.05 as indicated by one-way ANOVA followed by Holm–Sidak method.

To evaluate if treatment with an insulin sensitizer, such as pioglitazone, could restore muscle-specific miRNAs, we treated DIO mice with pioglitazone 35 mg/kg/day. Pioglitazone treatment of HFD for 15 days partially restored insulin sensitivity as measured by the Kitt (C: 6.3 ± 0.7%/min vs. H: 2.7 ± 0.3%/min vs. HP: 4.8 ± 0.6%/min, *p* < 0.05). Regardless of improving insulin sensitivity, pioglitazone could not restore miR-1a, miR-133a, or miR-206 levels in soleus muscles (Figure 5).

## Discussion

Muscle-specific miRNAs (e.g., miR-1a, miR-133a, and miR-206) have been reported to play critical role in myogenesis. MyomiRs have been shown to target IGF-1 signaling pathway, which is associated with insulin response in skeletal myocytes. Evidence is presented herein that miRNAs may be markers of the evolution of insulin resistance in soleus muscle.

Glucose intolerance and IR in mice exposed to an HFD develops within 3 days on an HFD but does not worsen significantly, even after 12 weeks on an HFD (30). Regardless of hyperinsulinemia, IR, and glucose intolerance observed in 4 weeks of HFD, the onset of IR in skeletal muscle of DIO mice was observed only after 8 weeks with impact on skeletal muscle mass loss. In line with unaltered glucose metabolism in soleus muscles after 4 weeks in HFD, a recent work has shown the maintenance of muscle insulin sensitivity in mice fed with HFD for 3 weeks (31). After 5 weeks in HFD, an impaired insulin-mediated glucose uptake was observed in DIO mice (32).

Mattson’s group (33) showed that control laboratory mice are sedentary, glucose intolerant, and obese as compared to wild mice. Researchers have shown reducing daily food intake or providing exercise training results in increased plasma adiponectin and reduced insulin levels (34) and increases whole body insulin sensitivity mainly related to raised skeletal muscle glucose oxidation (35) in *ad libitum* fed and sedentary rodents. Thus, rodents housed under standard laboratory conditions are relatively insulin resistant (33), as observed herein after the 12-week period. Corroborating with these results, using a mice-to-human age map (36), for non-insulin dependent diabetes mellitus, the mouse age of 140 days (20-week-old) would be mapped to the 55-year-old in humans (*r*^2^ = 0.647); a high risk age for type 2 diabetes mellitus (37, 38).

Impaired insulin action or release impacts negatively on skeletal muscle health as this tissue is the largest body site for glucose consumption. Diabetic myopathy, characterized by reduced physical capacity, strength, and skeletal muscle mass is a common complication of diabetes. Our data show a reduction in skeletal muscle mass, specifically gastrocnemius muscle, exactly at the same time IR in skeletal muscle is established. A recent work on skeletal muscle regeneration demonstrated a delayed muscle repair process in DIO mice and an impaired activation of muscle satellite cells (39), which might contribute to sarcopenia related to type 2 diabetes.

There is cumulative evidence that miRNAs are associated with the development of diabetic complications (40). We evaluated the evolution of the expression of miR-1a, miR-133, and miR-206 in mice fed a HFD for 4, 8, or 12 weeks. A reduction in miR-1a expression in soleus of HFD-fed animals was observed, and, particularly, miR-133a was upregulated after 12 weeks of HFD compared to control mice. Interestingly, miR-1a expression, together with other variables, appears to account for fasting glycemia increments. Others have also shown a role of myomiRs in IR. Downregulation of miR-1, miR-206, and miR-133 levels has been reported in white adipocytes (41) and in gastrocnemius muscle (42) of DIO mice and in *vastus lateralis* (43) and plasma (44, 45) of type 2 diabetic patients.

Treatment with pioglitazone did not restore miRNAs expression in soleus muscle of HFD mice, despite the fact it increased insulin sensitivity. Not surprisingly, treatment with pioglitazone did not restore microRNAs expression in soleus muscle of HFD mice, despite the fact it increased insulin sensitivity. Agonists of peroxisome proliferator-activated receptor γ (PPARγ), such as pioglitazone, has shown to significantly inhibit myotube formation of C2C12 myoblasts, whereas GW9662, a PPARγ antagonist, increased the number and length of myotubes (46). In addition, the *in vitro* ectopic expression of PPARγ in C2C12 cells induced their transdifferentiation into adipocytes (47). This may be the reason pioglitazone (3 mg/kg) treatment of rats for 8 weeks induced fat accumulation in oxidative and glycolytic skeletal muscles (48).

miR-1a, miR-133a, and miR-206 are specifically induced during myogenesis (49) and myogenic transcription factors, such as MyoD, Myf5, Myog, and Mef2, mediate upregulation of these miRNAs (49–51). Alternatively, miRNAs repress proteins important for muscle differentiation such as HDAC4, a negative regulator of myogenesis, myostatin, and follistatin-like 1 (52, 53). Consistent with miR-1a reduction, soleus muscle from HFD-fed mice showed lower expression of *Myf5*, a transcription factor essential for miR-1a upregulation compared to control-fed mice (51). Zhou et al. (54) also found lower expression of β-catenin-related genes, *Myf5, MyoD*, and *Irs-1*, in skeletal muscle of DIO rats when compared with rats fed with a HFD that remained lean (obesity-resistant). The authors of this study postulated a cross talk between Wnt/β-catenin, and insulin sensitivity markers exists in skeletal muscle of obese rats. We also observed an increase of *Hdac4* and a decrease in follistatin (*Fst*) from 4 to 12 weeks of HFD, but only after 12 weeks of HFD feeding, *Hdac4* was different from control mice. Follistatin controls skeletal muscle development through antagonizing the myogenic inhibitor myostatin (55), and Sun et al. (29) have shown miR-1 suppresses HDAC4 and subsequently upregulates the production of follistatin, stimulating skeletal myocyte fusion *in vitro* and *in vivo*.

Among putative miR-1a, miR-206, and miR-133a targets, IGF-1, IGF-1R, and FSTL1 were already validated (15, 16, 52). Feedback circuits in which miR-1a and miR-133a control the level of IGF-1, that in turn regulates miR-1a and miR-133a, have been described during skeletal muscle development (15, 16). *IGF-1* mRNA and IGF-1 signaling *via* Igf-1R were impaired in soleus muscle of 12 weeks HFD-fed mice compared to that from control mice at the same time miR-133a expression was upregulated; suggesting miR-133a is mainly involved with IGF-1/IGF-1R/PI3K/AKT/Mtor signaling pathway. Ectopically overexpression of miR-133 in C2C12 cells reduces IGF-1-stimulated phosphorylation of Akt at Serine-473, the Akt activation site (15), which mediates IGF-1 anabolic and anti-catabolic effects due *via* mTOR by inactivation of Foxo3. A significant reduction in PI3K activity, an upstream target that activates AKT, was observed in skeletal muscle of obese mice, which was associated to decreased myofiber growth and collagen deposition (56). In addition, Brown et al. (57) have shown diet-induced obesity reduced Igf-1 and Igf-1 signaling in skeletal muscle of mice before and after 28 days post-injury with bupivacaine injection, impairing skeletal muscle regenerative response. Therefore, our data suggest increased miR-133a expression may be responsible for impaired induction of protein synthesis signaling in obese mice. miRNA miR-1a did not present a time and diet interaction effect; however, miR-1a expression is lower in HFD mice compared to control and is reduced over the time. Therefore, probably, miR-1a contributes for reduced myogenesis in HFD animals. As well as this, miR-1a was correlated to glycemia.

**Figure 6:**
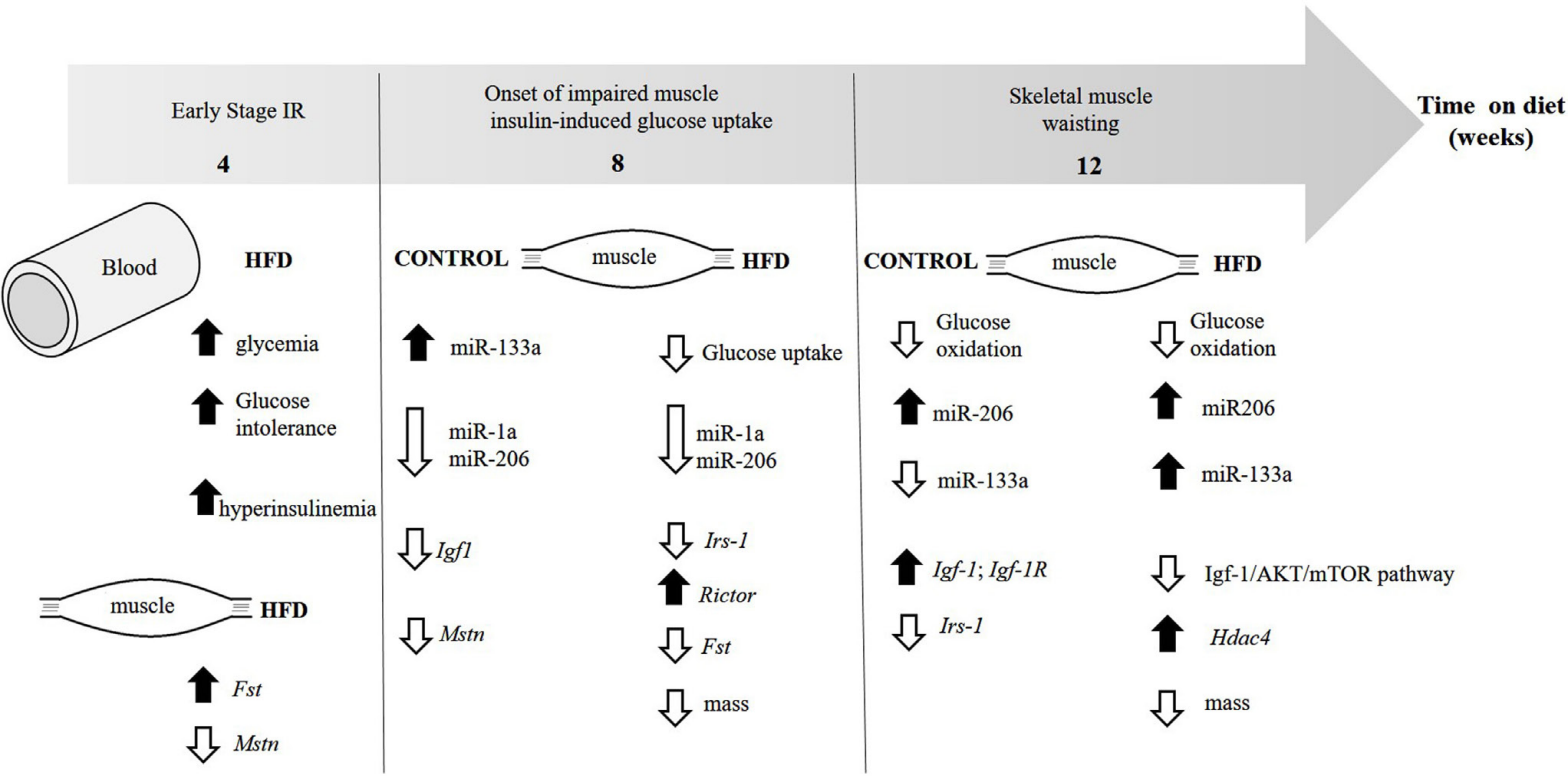
**Evolution of the changes in skeletal muscle and insulin sensitivity in diet-induced obese mice**. The figure illustrates the time each parameter first appear.

In conclusion, as summarized in Figure 6, we provided for the first time data indicating decreased myogenesis in oxidative skeletal muscle of insulin-resistant mice due to dysregulation in expression of myomiRs, mainly miR-1a and miR-133a. Type 2 diabetes negatively affects skeletal muscle function and mass, which may be a result of increased miR-133a expression together with low Igf-1 in skeletal muscle. While miR-1a may be used as a biomarker for the manifestation and progression of diabetes, and lately glycemic control, it has been found dysregulated in plasma of diabetic subjects (44, 45). Therefore, miR-1a may be a candidate for an early marker for increments on glycemia and miR-133a for skeletal muscle wasting in diabetic subjects.

## Author Contributions

Conceived and designed the research: AR. Acquired, analyzed, or interpreted data: FF, MM, AG, AR, and BC. Wrote the manuscript: FF, RC, and AR.

## Conflict of Interest Statement

The authors declare that the research was conducted in the absence of any commercial or financial relationships that could be construed as a potential conflict of interest. The reviewer (CS) and handling Editor declared their shared affiliation, and the handling Editor states that the process nevertheless met the standards of a fair and objective review.
